# Supplementary material for: Application of Crosslinked Polybenzimidazole-Poly(Vinyl Benzyl Chloride) Anion Exchange Membranes in Direct Ethanol Fuel Cells
Source: Membranes (Basel). 2020 Nov 17;10(11):349. doi: 10.3390/membranes10110349 (PMC7698426; doi:10.3390/membranes10110349)
Supplement: Supplementary file 1 [file membranes-10-00349-s001.pdf]

membranes

Supplementary Materials

Application of Crosslinked Polybenzimidazole-Poly(Vinyl Benzyl Chloride) Anion Exchange Membranes in Direct Ethanol Fuel Cells

Daniel Herranz [1,*], Roxana E. Coppola [2], Ricardo Escudero-Cid [1,3], Kerly Ochoa-Romero [1], Norma B. D'Accorso [4,5], Juan Carlos Pérez-Flores [6], Jesús Canales-Vázquez [6], Carlos Palacio [7], Graciela C. Abuin [2] and Pilar Ocón [1]

1. Departamento de Química Física Aplicada, Universidad Autónoma de Madrid, C/Francisco Tomás y Valiente 7, 28049 Madrid, Spain; ricardo.escudero@campusviu.es (R.E.-C.); kerly.ochoa@estudiante.uam.es (K.S.-O.); pilar.ocon@uam.es (P.O.)
2. Instituto Nacional de Tecnología Industrial (INTI), Departamento de Almacenamiento de Energía, Av. General Paz 5445, B1650KNA San Martín, Argentina; rcoppola@inti.gob.ar (R.E.C.); gabuin@inti.gob.ar (G.C.A.)
3. Departamento de Educación, Universidad Internacional de Valencia (VIU), C/Pintor Sorolla 21, 46002 Valencia, Spain
4. Departamento de Química Orgánica, Facultad de Ciencias Exactas y Naturales, Universidad de Buenos Aires, C1428, Buenos Aires, Argentina; norma@qo.fcen.uba.ar
5. CONICET—Universidad de Buenos Aires, Centro de Investigaciones en Hidratos de Carbono (CIHIDECAR), C1428, Buenos Aires, Argentina
6. Instituto de Investigación de Energías Renovables, Universidad de Castilla-La Mancha, Calle Investigación 1, Edif. 3. 02071 Albacete, Spain; JuanCarlos.PFlores@uclm.es (J.C.P.-F.); jesus.canales@uclm.es (J.C.-V.)
7. Departamento de Física Aplicada, Universidad Autónoma de Madrid, C/Francisco Tomás y Valiente 7, 28049 Madrid, Spain; carlos.palacio@uam.es

* Correspondence: daniel.herranz@uam.es

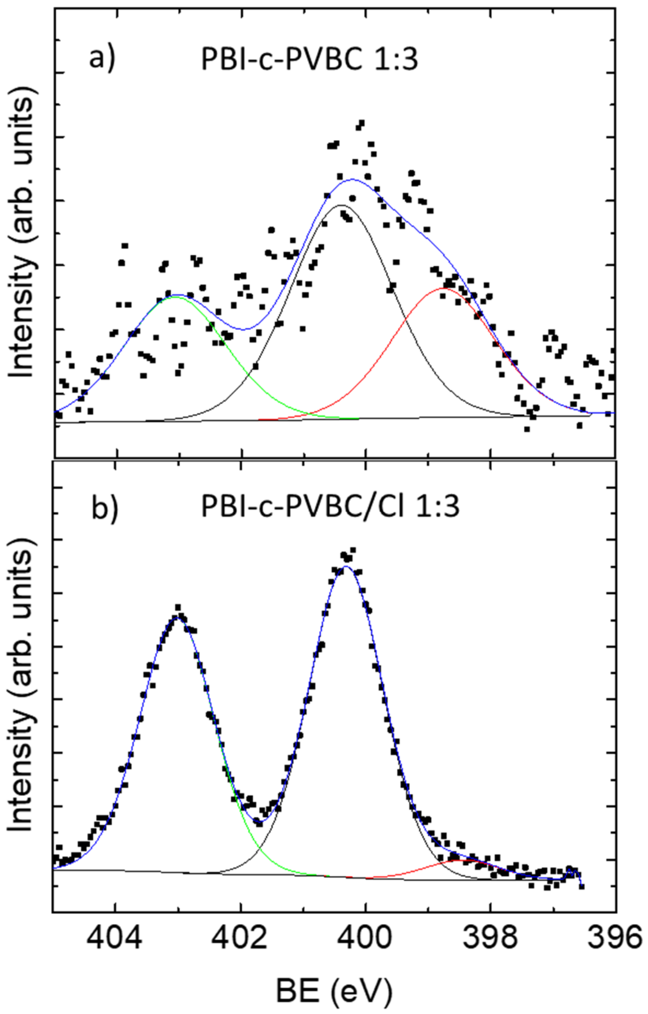

Figure S1. XPS N 1s peaks of (**a**) PBI-c-PVBC 1:3 membrane (crosslinked) and (**b**) PBI-c-PVBC/Cl 1:3 membrane (after quaternization with DABCO).

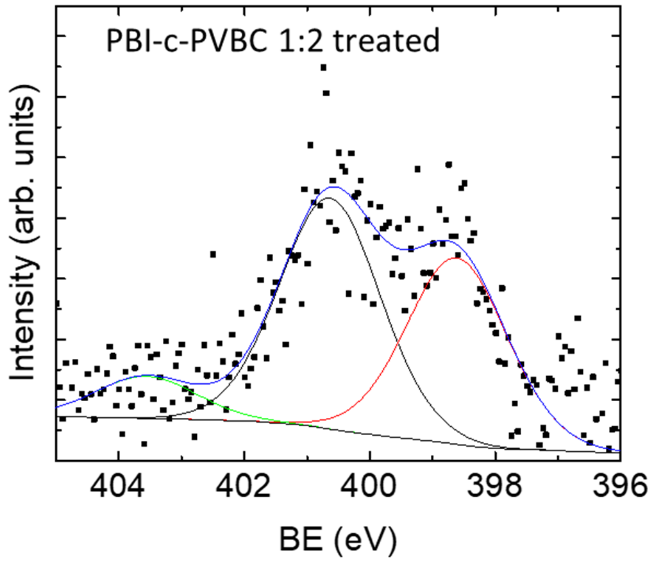

Figure S2. XPS N 1s peaks of PBI-c-PVBC 1: membrane (crosslinked) after immersion in EtOH at 60 °C for 3 days (in absence of DABCO).

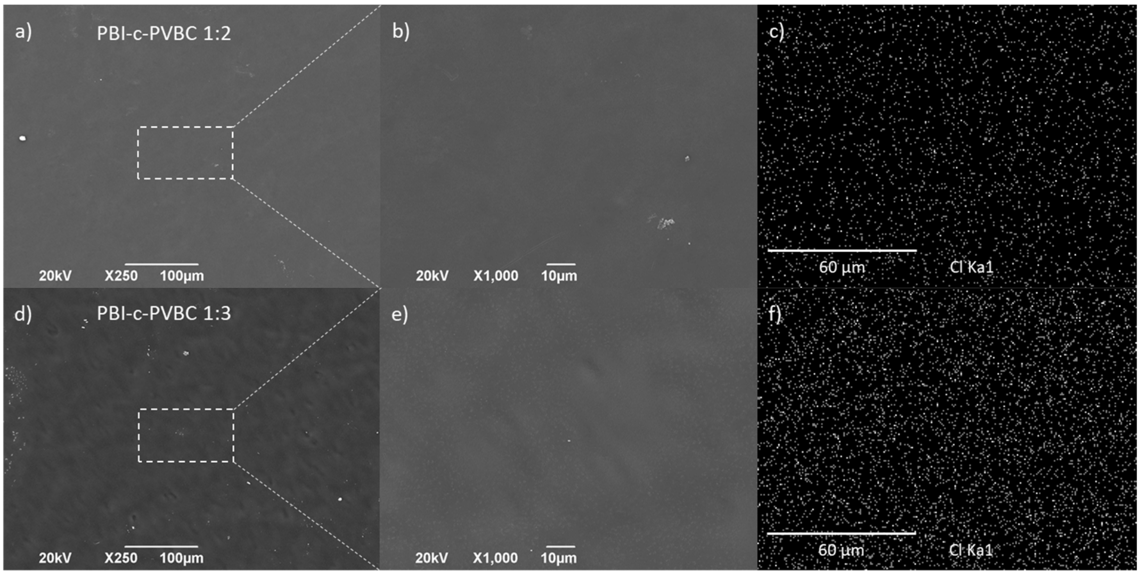

Figure S3. Surface (**a**) and (**d**) SEM micrographs; (**b**) and (**e**) detailed inset and (**c**) and (**f**) corresponding Cl EDX mapping of PBI-c-PVBC 1:2 and 1:3 membranes, respectively.

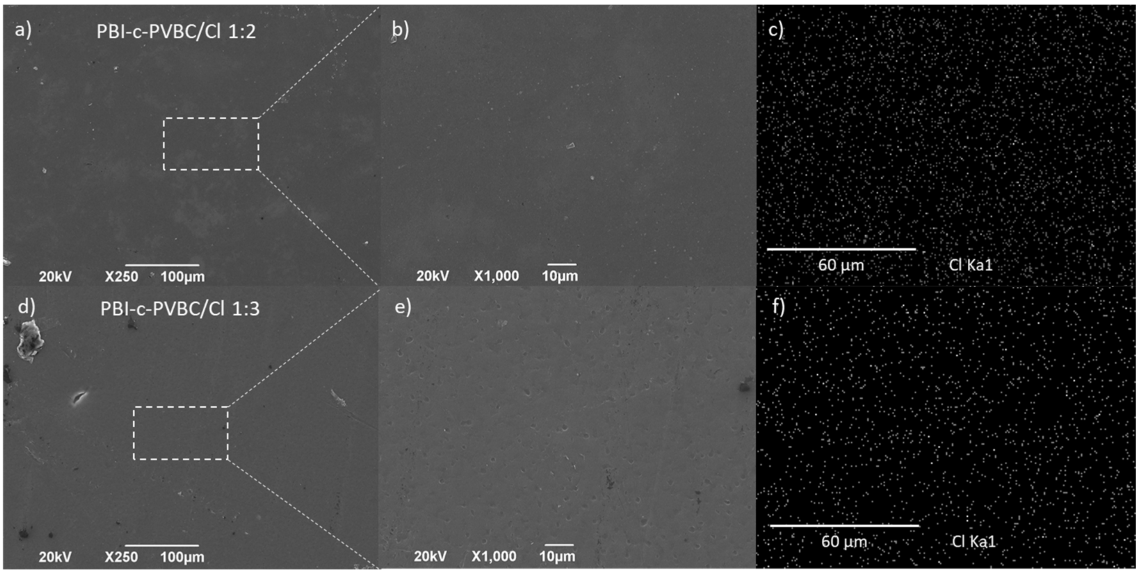

Figure S4. Surface (**a**) and (**d**) SEM micrographs; (**b**) and (**e**) detailed inset and (**c**) and (**f**) corresponding Cl EDX mapping of PBI-c-PVBC/Cl 1:2 and 1:3 membranes, respectively.

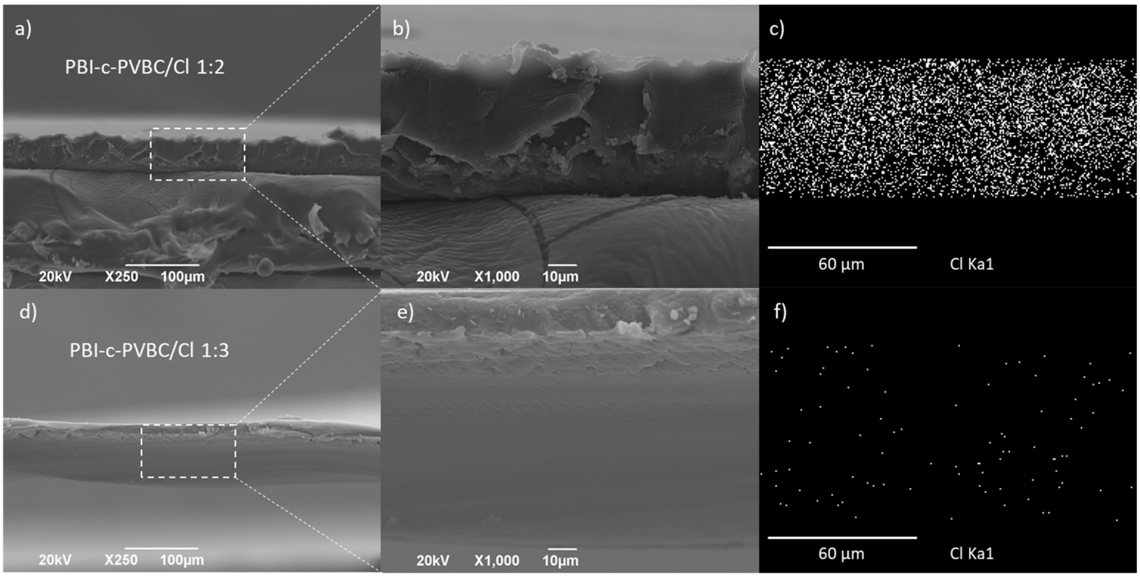

Figure S5. Cross-section (**a**) and (**d**) SEM micrographs; (**b**) and (**e**) detailed inset and (**c**) and (**f**) corresponding Cl EDX mapping of PBI-c-PVBC/Cl 1:2 and 1:3 membranes, respectively. The membrane of 1:3 ratio shows a different aspect and chloride mapping because the picture was out of focus.

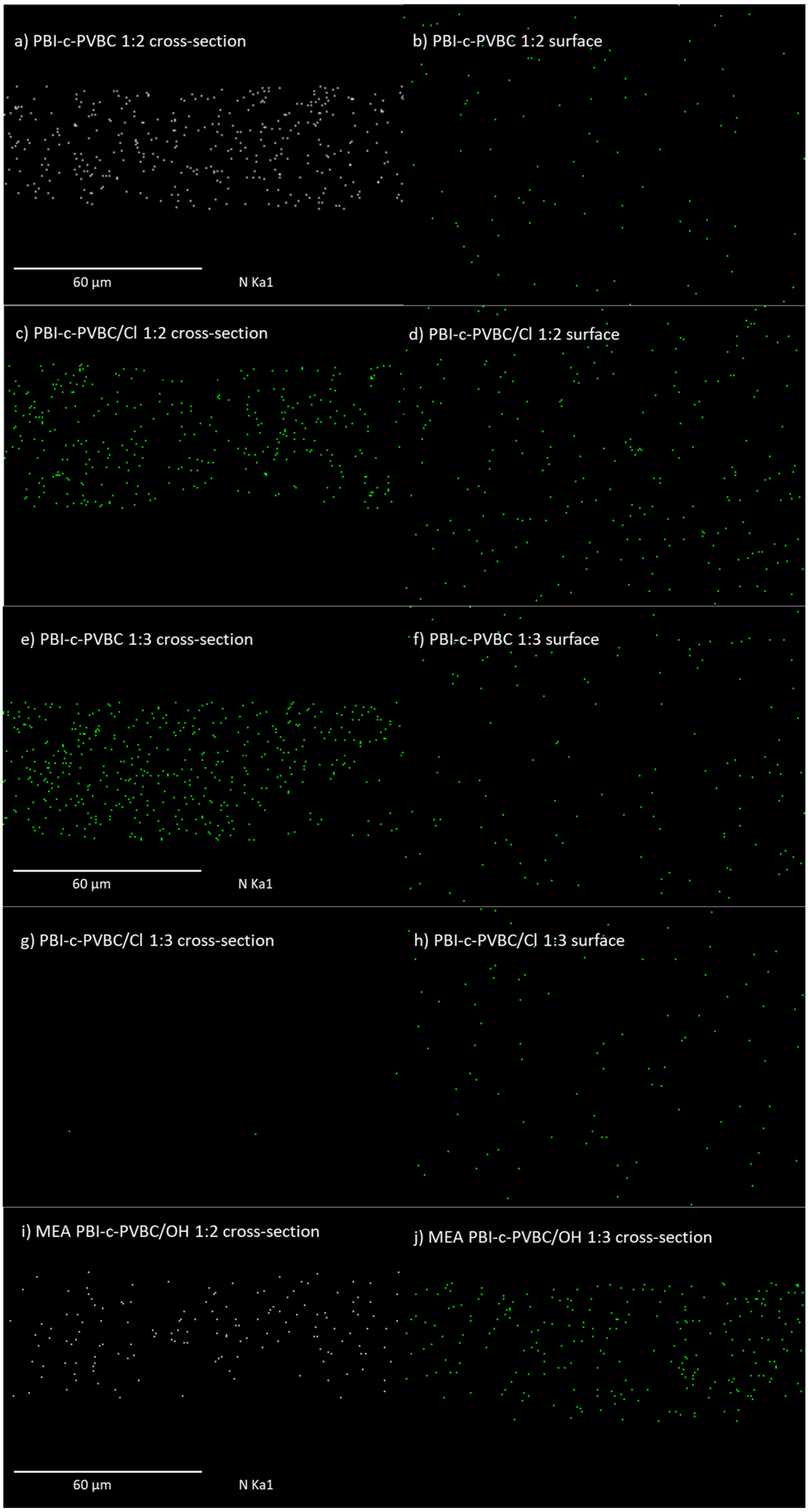

Figure S6. EDX N mapping of the surface and cross-section of crosslinked (PBI-c-PVBC), quaternized (PBI-c-PVBC/Cl) and after fuel cell test (MEA PBI-c-PVBC/OH) samples. All were done with ×1000 magnifications.

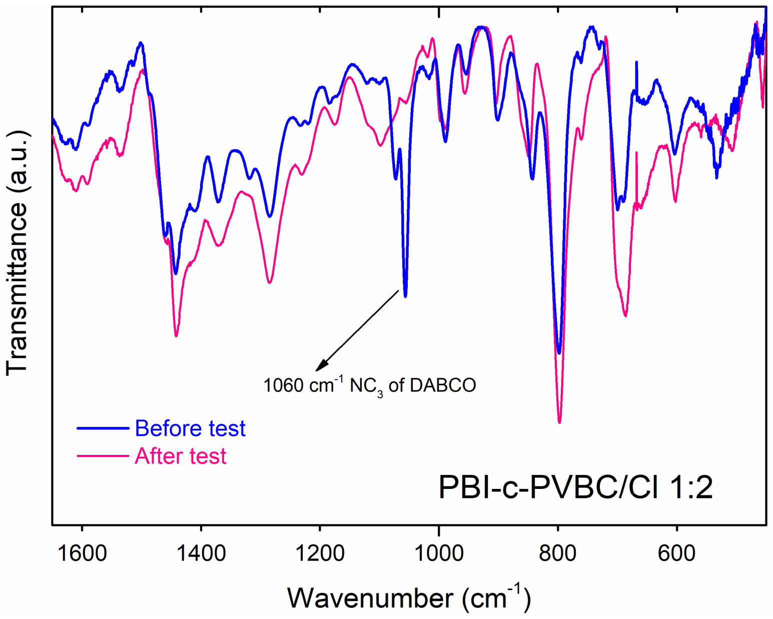

Figure S7. FT-IR spectra of the PBI-c-PVBC/Cl 1:2 membrane before and after the accelerated oxidative degradation durability test.
